# Supplementary material for: Computed Tomography-Based Sarcopenia and Pancreatic Cancer Survival—A Comprehensive Meta-Analysis Exploring the Influence of Definition Criteria, Prevalence, and Treatment Intention
Source: Cancers (Basel). 2025 Feb 11;17(4):607. doi: 10.3390/cancers17040607 (PMC11853262; doi:10.3390/cancers17040607)
Supplement: Supplementary file 1 [file cancers-17-00607-s001.zip › Supplementary File S2. Review protocol.pdf]

Internal Protocol for systematic review and meta-analysis on Sarcopenia and Survival Outcomes in Pancreatic Cancer Measured by Computed Tomography

Version 1.0

Date: 22 April, 2024

1. Objective of the study

The aim of this meta-analysis is to assess the impact of sarcopenia, as measured by computed tomography (CT), on overall survival (OS) and progression-free survival (PFS) in patients with pancreatic cancer (PC). Additionally, the study will explore the influence of different sarcopenia measurement methods, cut-off values, and patient management approaches on these outcomes.

2. Research questions addressed

What is the effect of sarcopenia on overall survival (OS) and progression-free survival (PFS) in patients with pancreatic cancer, when assessed by CT?

How do the associations between sarcopenia and survival outcomes vary according to the method of sarcopenia assessment and the cut-off values used?

Does the oncological context (curative vs. palliative management) modify the impact of sarcopenia on survival outcomes?

3. Eligibility criteria

- **Types of studies**: Observational studies reporting hazard ratios (HRs) for overall survival (OS) and/or progression-free survival (PFS) in patients with pancreatic cancer stratified by sarcopenia status assessed through CT.
- **Population**: Adult patients diagnosed with pancreatic cancer (regardless of disease stage).
- **Intervention**: Assessment of sarcopenia using CT-based skeletal muscle mass or area (e.g., skeletal muscle index [SMI], total psoas area/volume [TPA/TPV]).
- **Outcomes**: Hazard ratios for OS and PFS based on sarcopenia status, with secondary exploration of subgroups by measurement method, cut-off value, and patient management (curative vs. palliative).
- **Language**: No language restrictions.
- **Date**: No restrictions on publication date.

- **Exclusion criteria**: Editorials, letters, conference abstracts, or studies without complete data on hazard ratios for survival outcomes.

4. Information sources and search strategy

PubMed, Web of Science, and EMBASE databases will be searched extensively to identify studies published from the inception of the databases to the date of the search.

Search terms related to "pancreatic neoplasia", "sarcopenia", "cachexia", and "body composition" will be used. References of all articles read in their entirety will also be reviewed to increase the sensitivity of the search.

5. Study selection

Two independent reviewers will evaluate the titles and abstracts of the identified studies to determine their eligibility. Studies that meet the inclusion criteria will be read in their entirety to confirm final eligibility. Any disagreement will be resolved by discussion or consultation with a third reviewer.

6. Data extraction

- **Study characteristics**: Author, year of publication, country, study design, number of participating institutions.
- **Patient characteristics**: Sample size, age, sex, and baseline characteristics of patients.
- **Sarcopenia assessment**: Sarcopenia measurement method (e.g., SMI, TPA/TPV), cut-off values used for males and females.
- **Cancer-related characteristics**: Type and stage of pancreatic cancer, and treatment intent (curative or palliative).

- **Survival outcomes**: Crude and adjusted hazard ratios (HRs) for overall survival (OS) and progression-free survival (PFS).

7. Subgroup analyses

Subgroup analyses will be conducted based on:

- **Method used to assess sarcopenia**: Skeletal muscle index (SMI), total psoas area (TPA), or other CT-based measurements.
- **Sarcopenia cut-off values**: Cut-off thresholds used to define sarcopenia (e.g., <50 cm²/m² vs. >50 cm²/m² for males).
- **Oncological management**: Curative intent (patients eligible for resection or other curative treatments) versus palliative management (patients with unresectable or metastatic disease).

8. Evaluation of the quality of the studies

The Newcastle-Ottawa Scale (NOS) will be used to assess the quality of the included studies, evaluating three categories: selection, comparability and outcome, with a maximum score of 9 stars.

9. Statistical analysis

A random-effects model will be used for the meta-analyses, incorporating the Hartung-Knapp (HK) adjustment for more conservative confidence intervals. The hazard ratio (HR) will be the effect measure for survival outcomes (OS and PFS). Heterogeneity across studies will be assessed using the I^2 statistic, with $I^2 > 75\%$ indicating substantial heterogeneity. Tau² (τ^2) will also be calculated to quantify between-study variance. The

prediction interval (PI) will be computed to provide an estimate of the range within which the true effect is likely to fall in a new study.
Leave-one-out sensitivity analyses will be conducted to evaluate the robustness of the results, and publication bias will be assessed using funnel plots and Egger's test.

10. Compliance with PRISMA guidelines

This meta-analysis will follow the guidelines established by the **PRISMA** (Preferred Reporting Items for Systematic Reviews and Meta-Analyses) guidelines for planning, conducting and reporting systematic reviews and meta-analyses to ensure the transparency, integrity and reproducibility of research, including systematic search, study selection, quality assessment and data analysis.
