# Supplementary figures and images for: Computed Tomography-Based Sarcopenia and Pancreatic Cancer Survival—A Comprehensive Meta-Analysis Exploring the Influence of Definition Criteria, Prevalence, and Treatment Intention
Source: Cancers (Basel). 2025 Feb 11;17(4):607. doi: 10.3390/cancers17040607 (PMC11853262; doi:10.3390/cancers17040607)

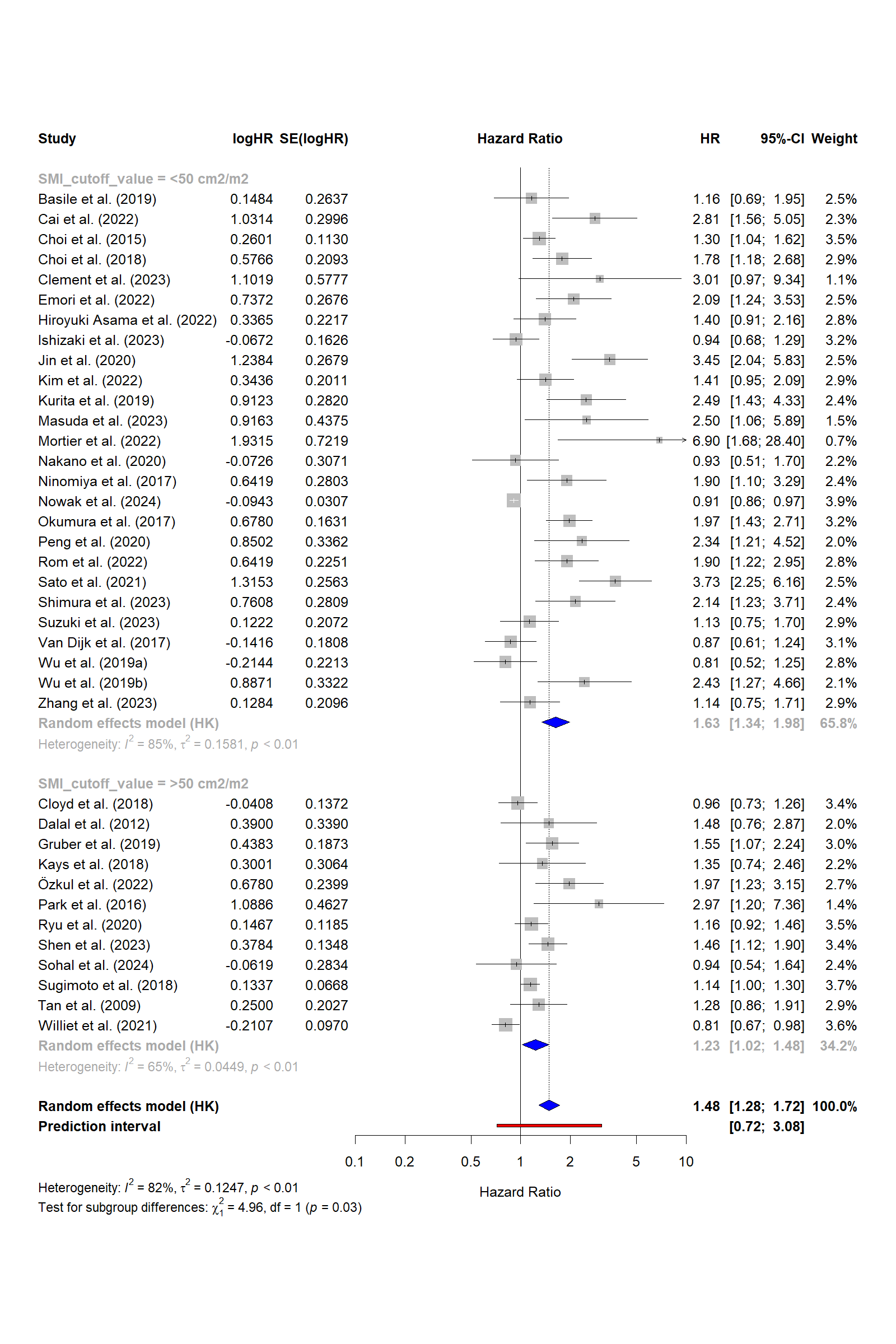

Supplement: Supplementary file 1 [file cancers-17-00607-s001.zip › Supplementary File S10. Forest_plot_OS_UNI_subgroup_SMI_cutoff.png]

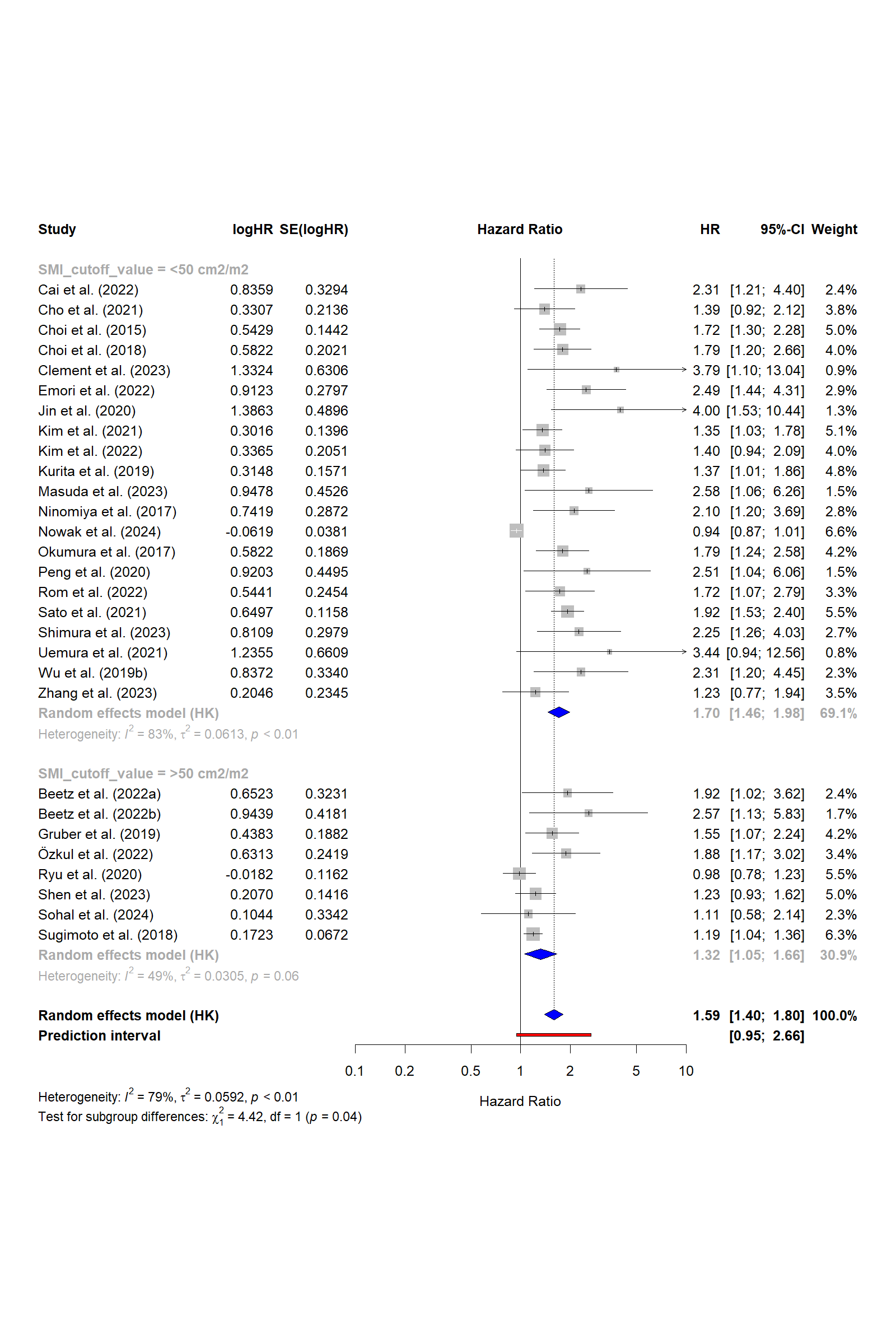

Supplement: Supplementary file 1 [file cancers-17-00607-s001.zip › Supplementary File S11. Forest_plot_OS_MULTI_subgroup_SMI_cutoff.png]

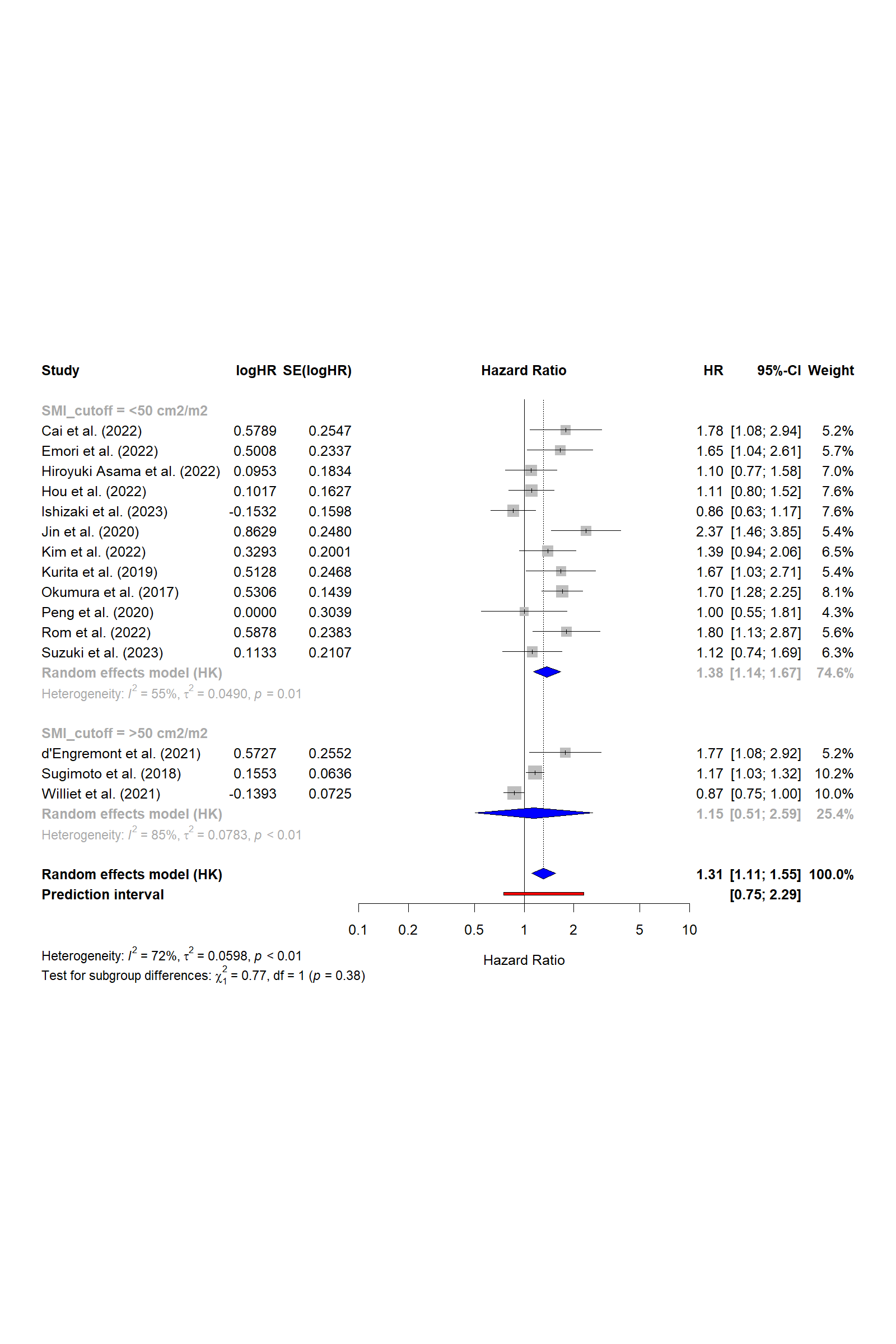

Supplement: Supplementary file 1 [file cancers-17-00607-s001.zip › Supplementary File S12. Forest_plot_PFS_UNI_subgroup_SMI_cutoff.png]

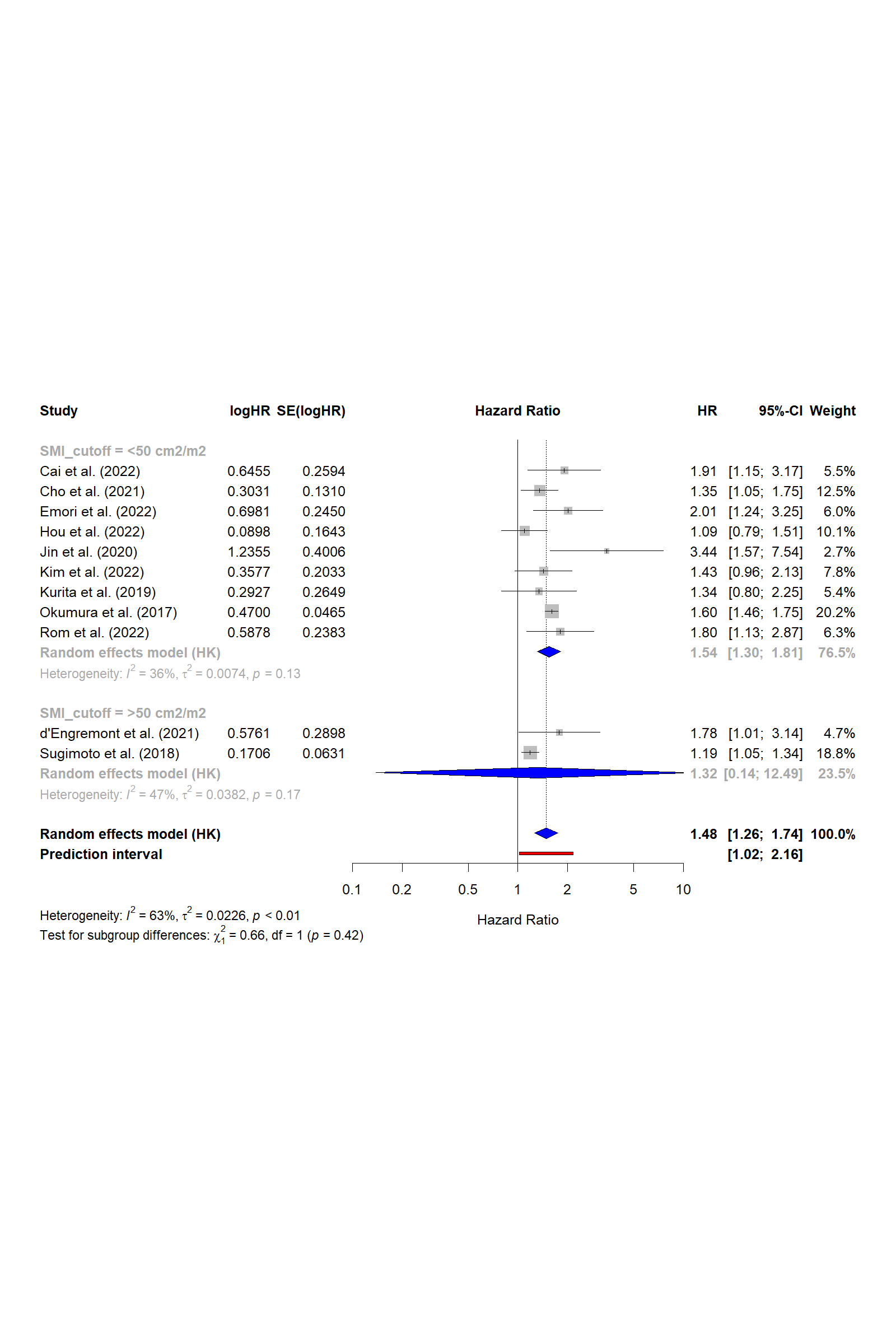

Supplement: Supplementary file 1 [file cancers-17-00607-s001.zip › Supplementary File S13. Forest_plot_PFS_MULTI_subgroup_SMI_cutoff.png]

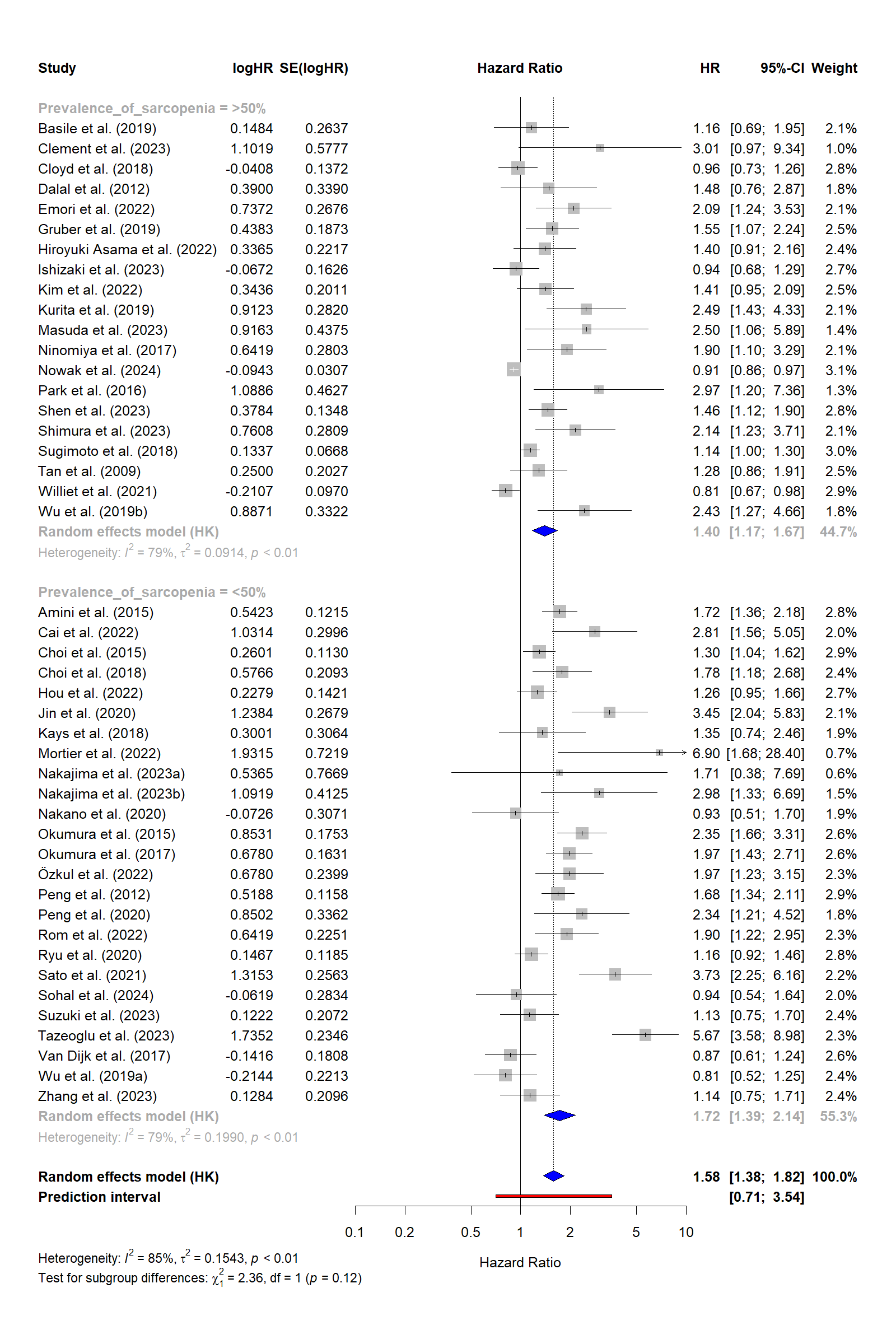

Supplement: Supplementary file 1 [file cancers-17-00607-s001.zip › Supplementary File S14. Forest_plot_OS_UNI_subgroup_prevalence_sarcopenia.png]

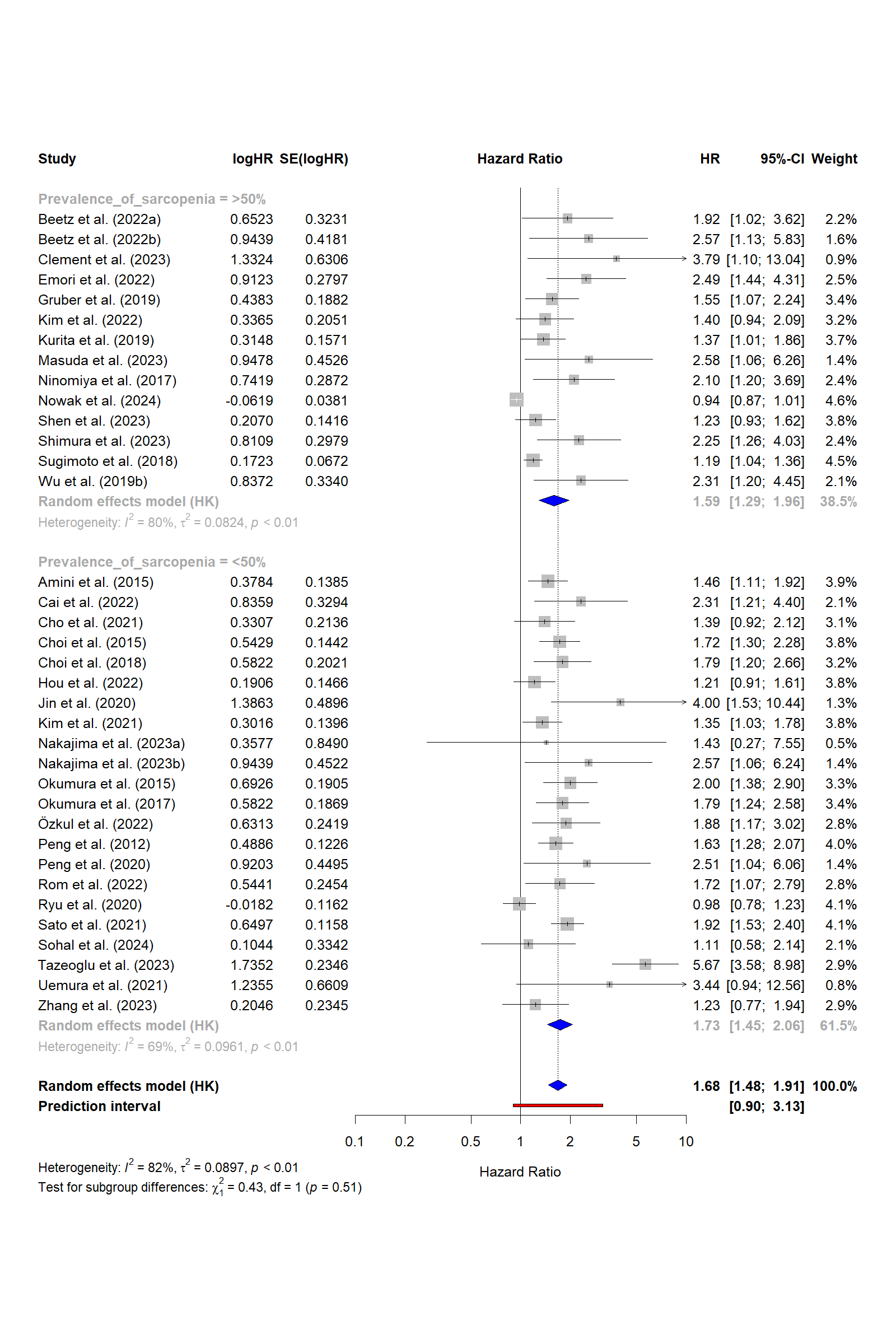

Supplement: Supplementary file 1 [file cancers-17-00607-s001.zip › Supplementary File S15. Forest_plot_OS_MULTI_subgroup_prevalence.png]

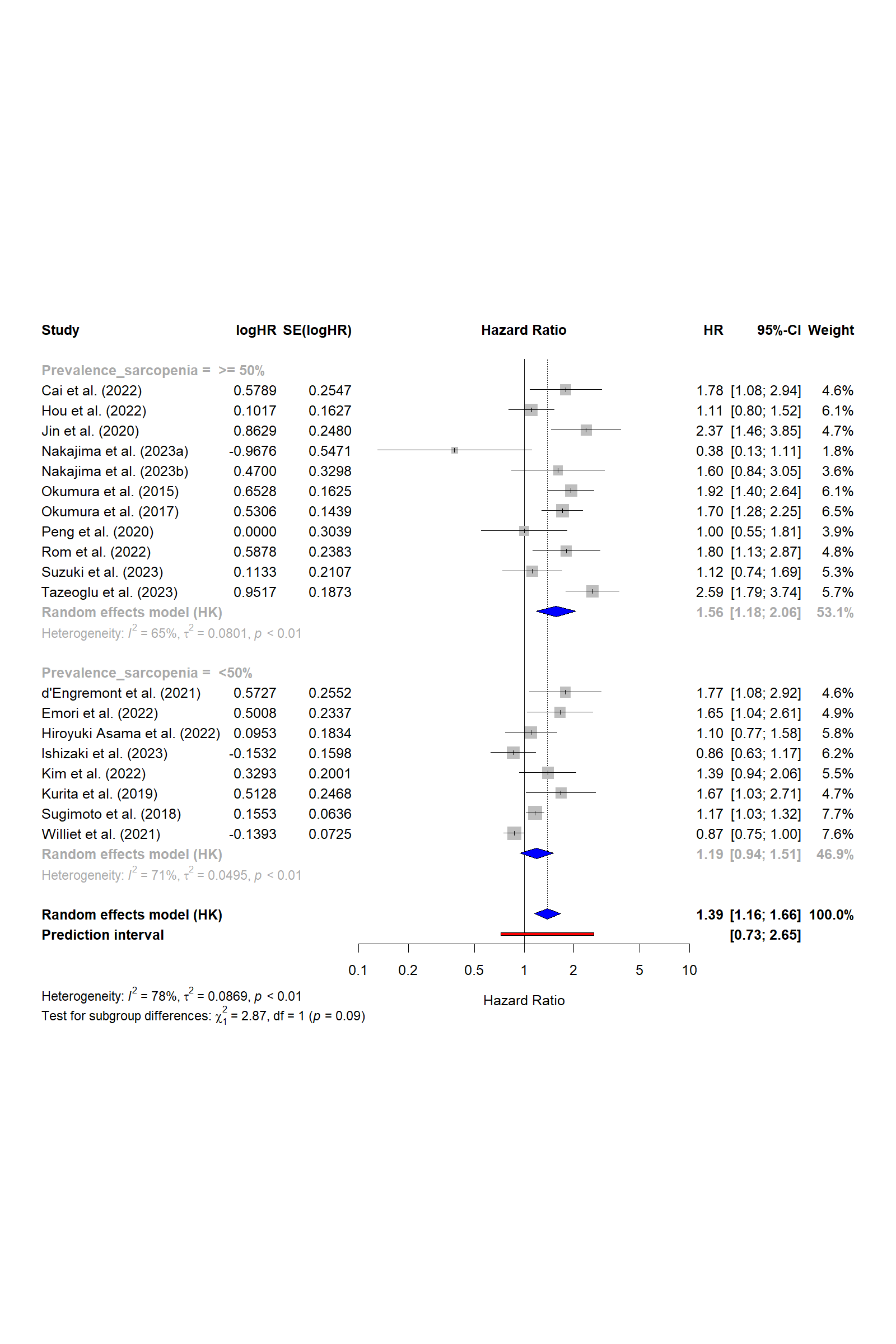

Supplement: Supplementary file 1 [file cancers-17-00607-s001.zip › Supplementary File S16. Forest_plot_PFS_UNI_subgroup_prevalence.png]

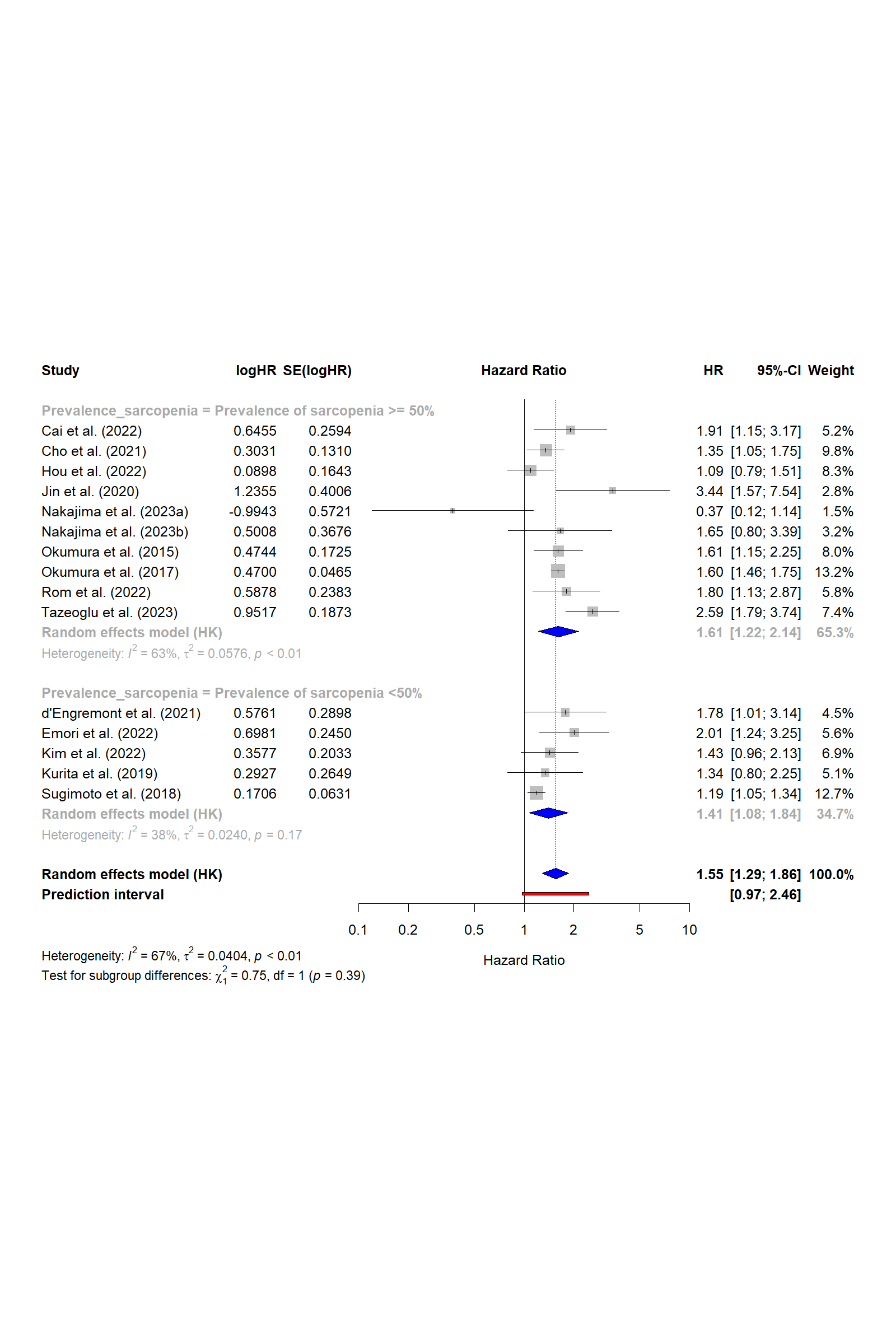

Supplement: Supplementary file 1 [file cancers-17-00607-s001.zip › Supplementary File S17. Forest_plot_PFS_MULTI_subgroup_prevalence.png]

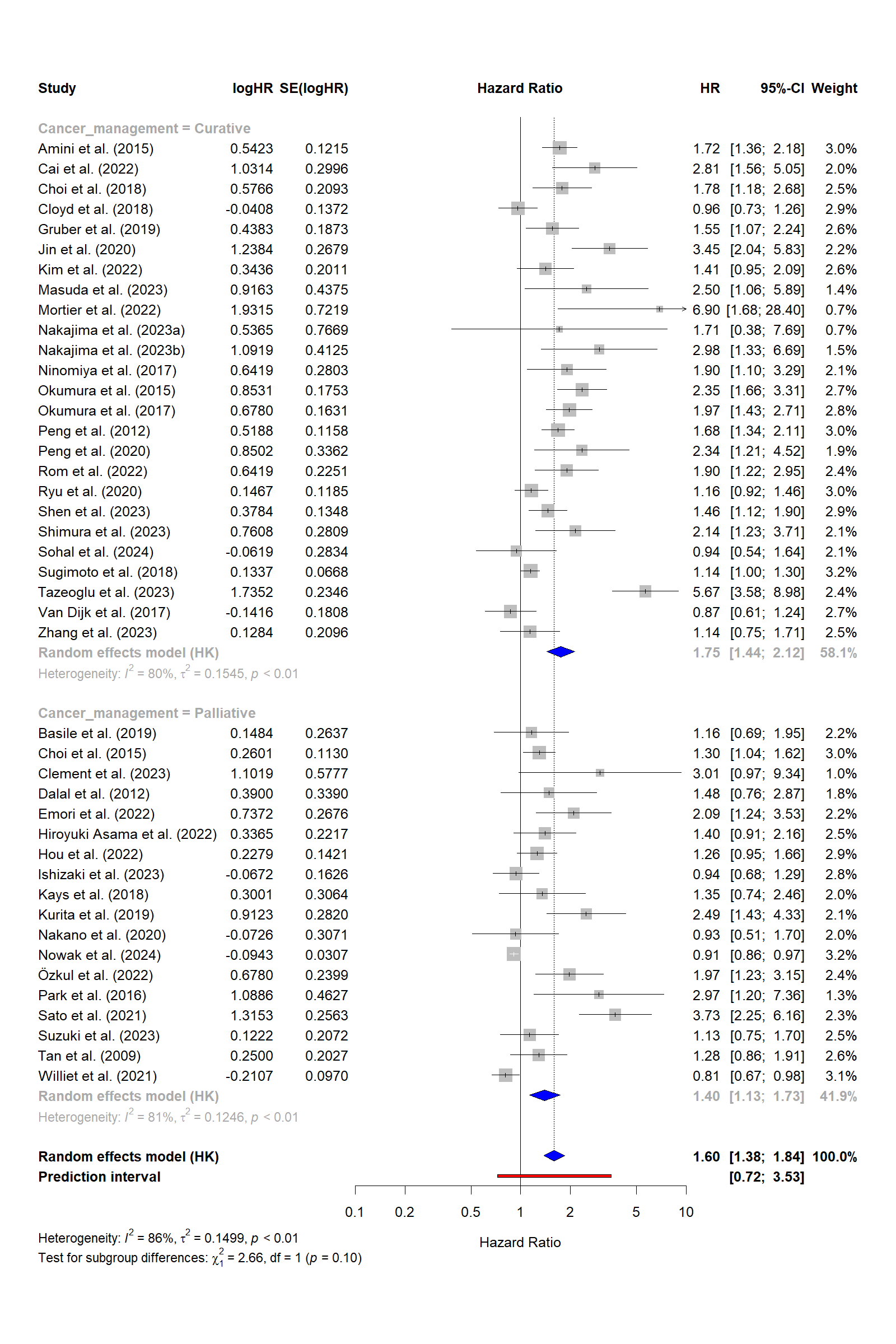

Supplement: Supplementary file 1 [file cancers-17-00607-s001.zip › Supplementary File S18. Forest_plot_OS_UNI_subgroup_treatment_intention.png]

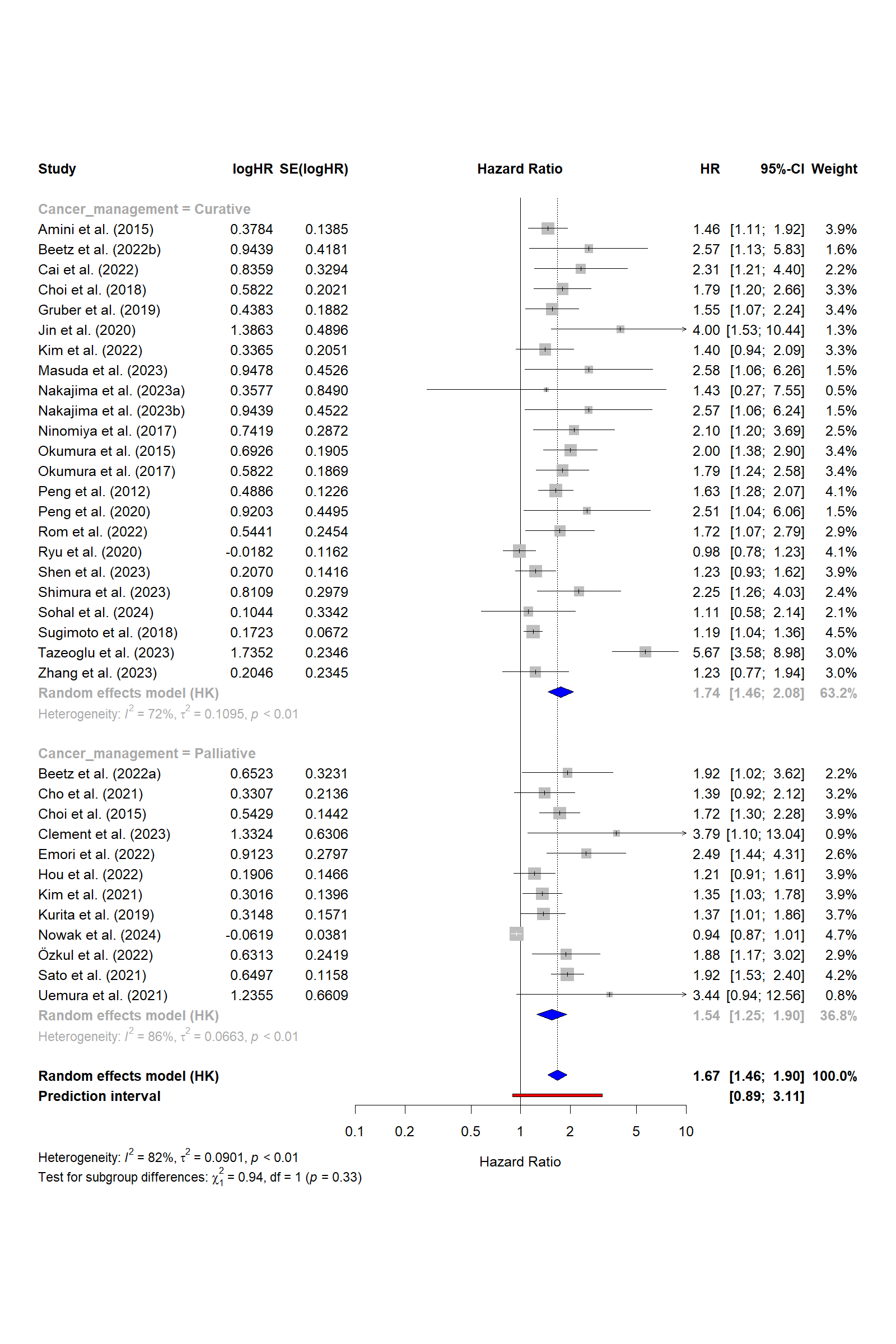

Supplement: Supplementary file 1 [file cancers-17-00607-s001.zip › Supplementary File S19. Forest_plot_OS_MULTI_subgroup_treatment_intention.png]

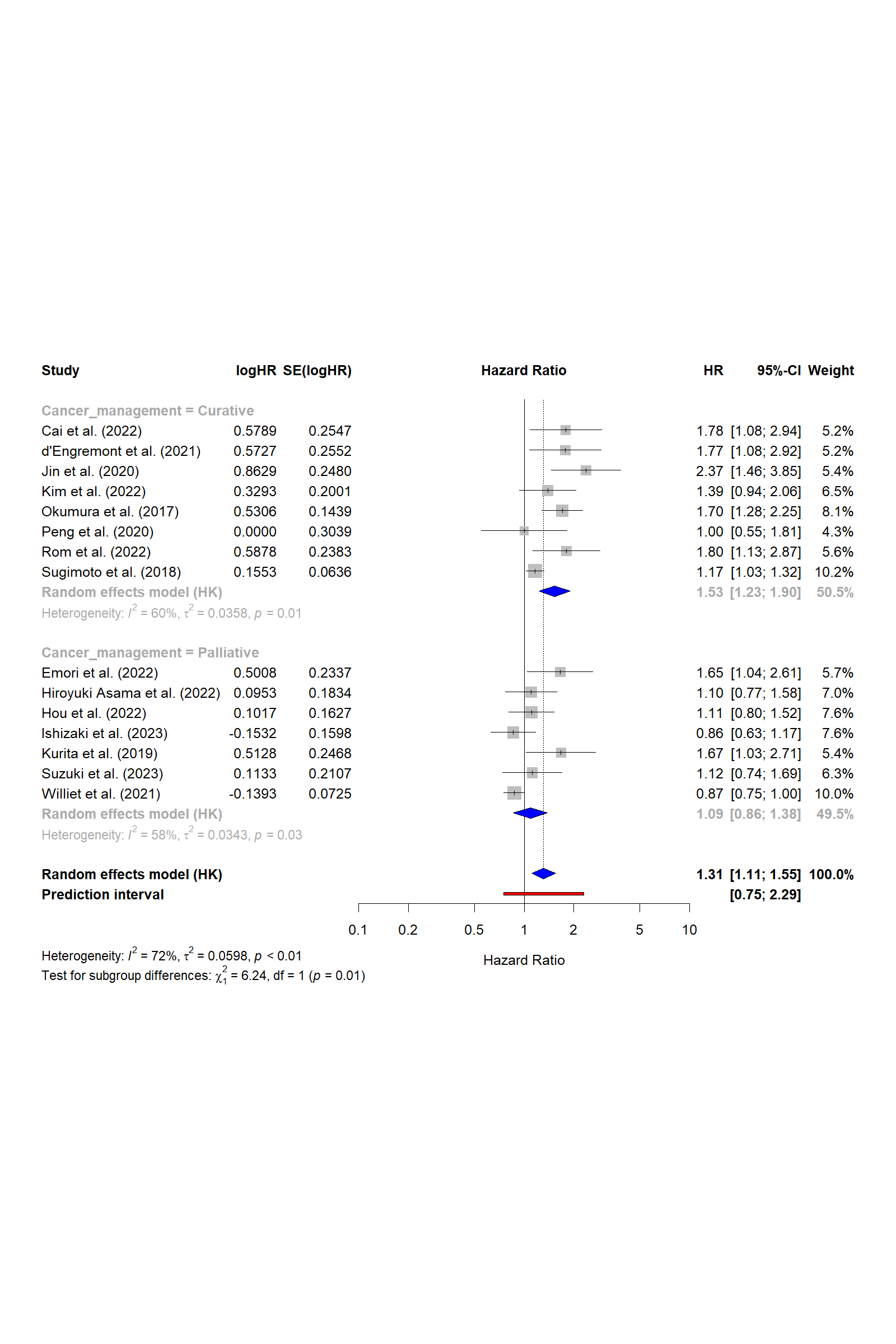

Supplement: Supplementary file 1 [file cancers-17-00607-s001.zip › Supplementary File S20. Forest_plot_PFS_UNI_subgroup_treatment_intention.png]

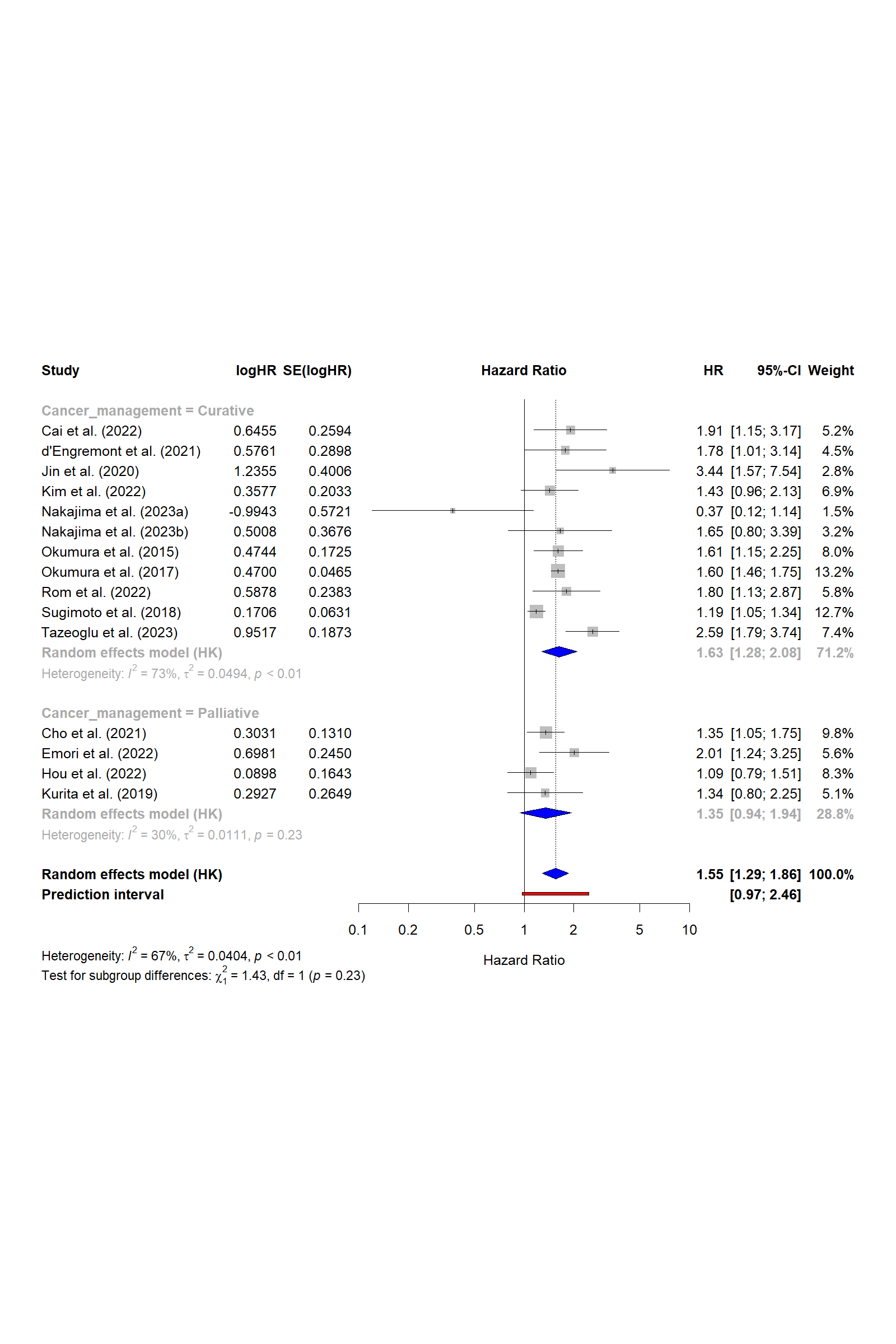

Supplement: Supplementary file 1 [file cancers-17-00607-s001.zip › Supplementary File S21. Forest_plot_PFS_MULTI_subgroup_treatment_intention.png]

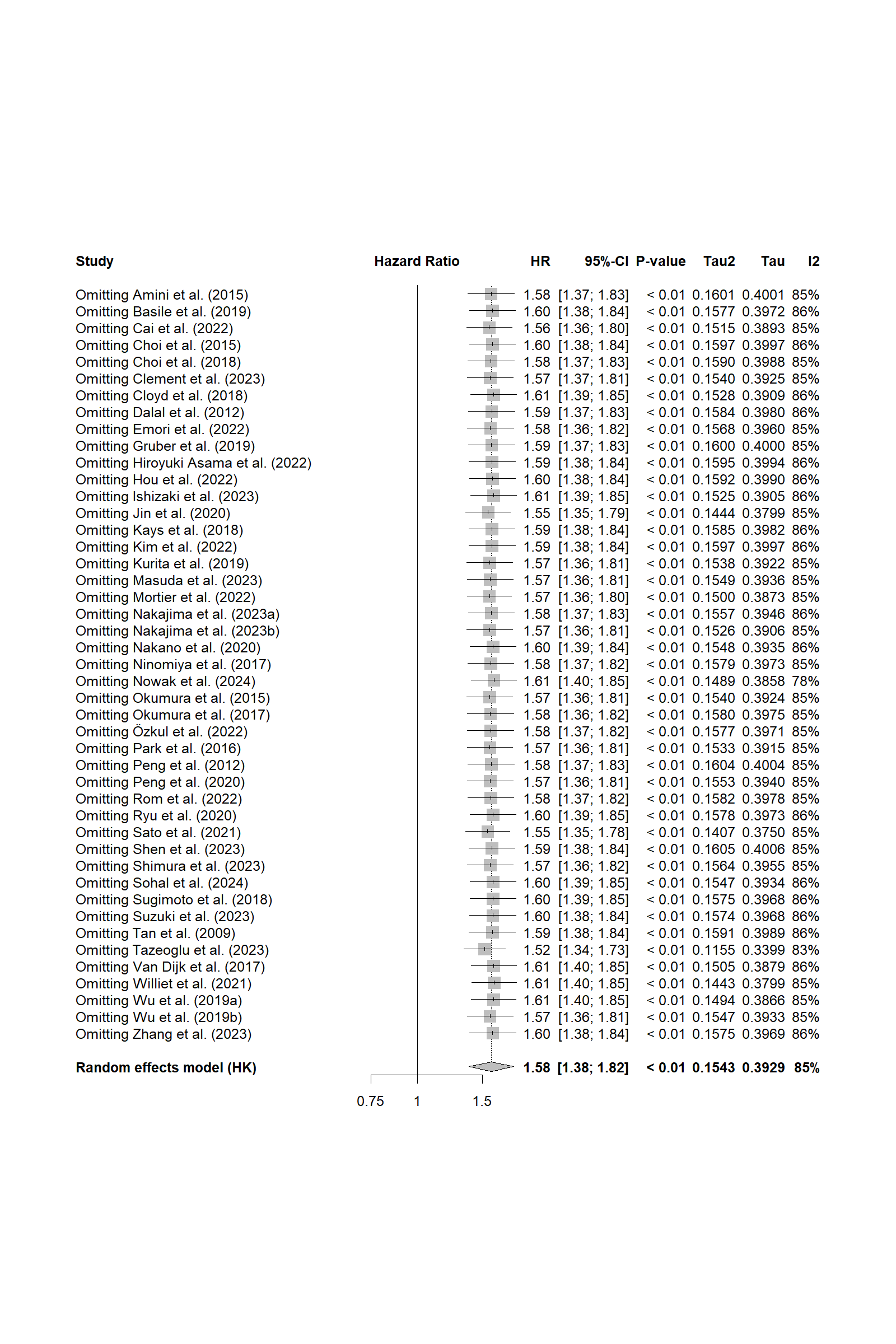

Supplement: Supplementary file 1 [file cancers-17-00607-s001.zip › Supplementary File S22. Sensitivity_analysis_OS_UNI.png]

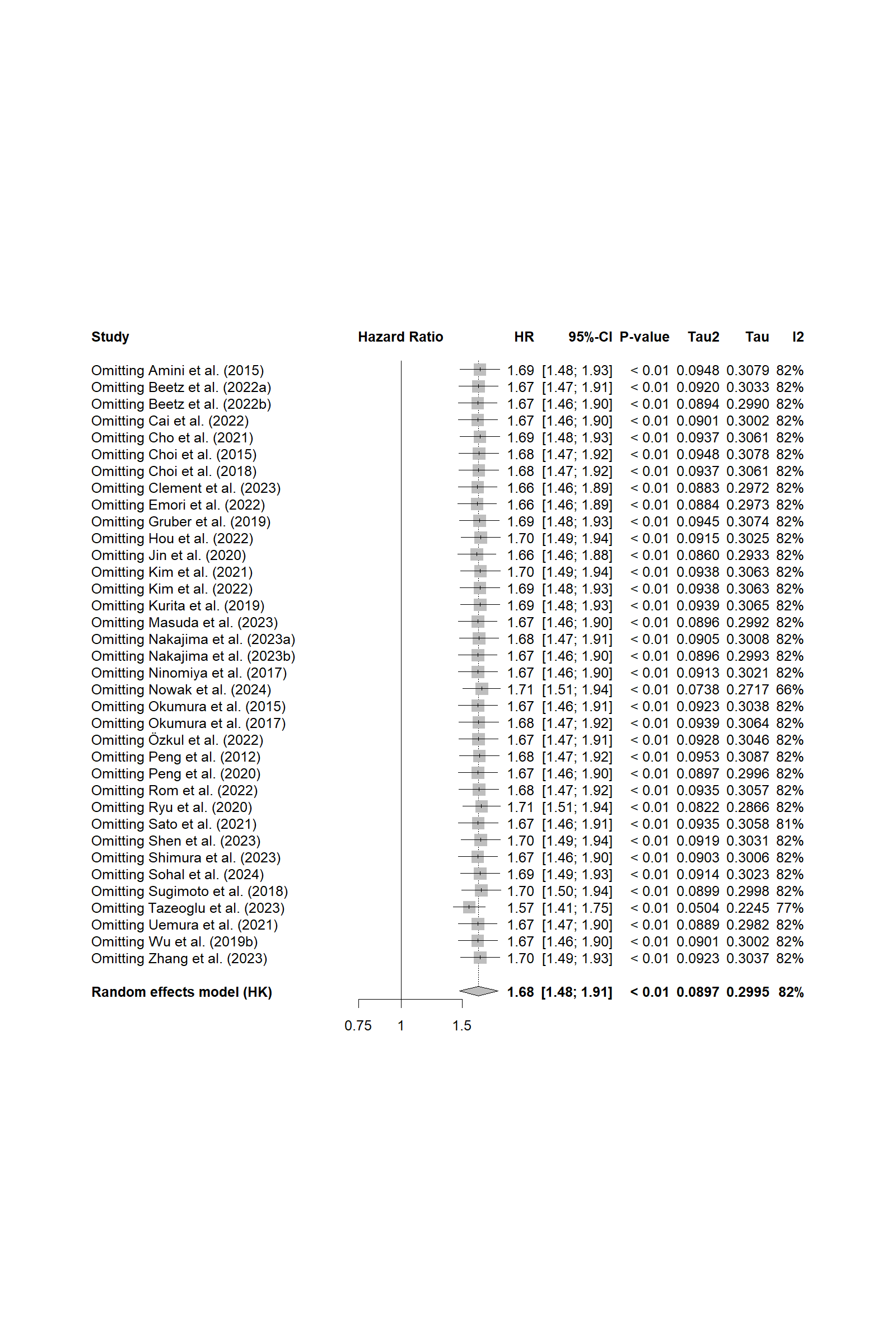

Supplement: Supplementary file 1 [file cancers-17-00607-s001.zip › Supplementary File S23. Sensitivity analysis OS_MULTI.png]

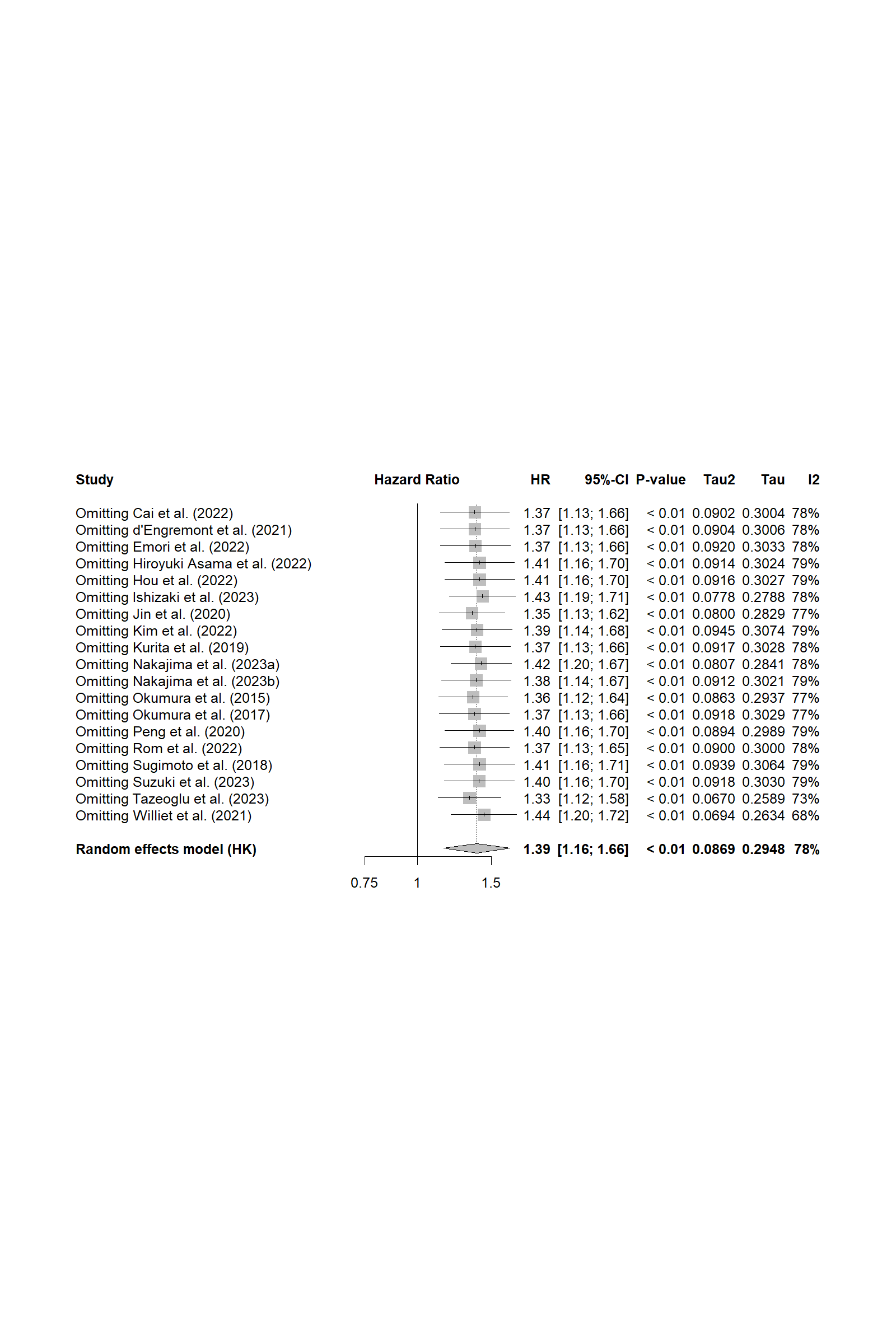

Supplement: Supplementary file 1 [file cancers-17-00607-s001.zip › Supplementary File S24. Sensitivity analysis PFS_UNI.png]

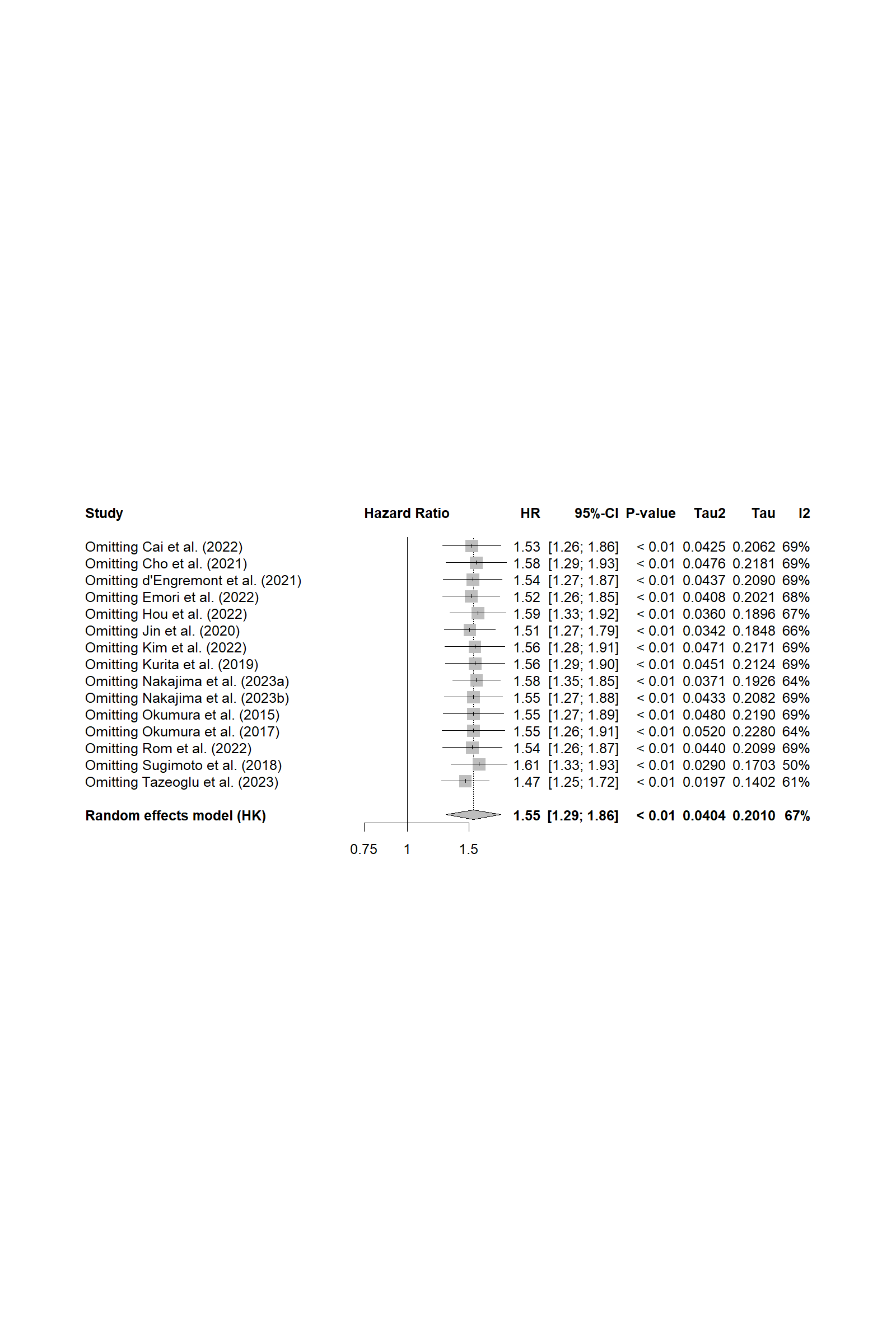

Supplement: Supplementary file 1 [file cancers-17-00607-s001.zip › Supplementary File S25. Sensitivity analysis PFS_MULTI.png]

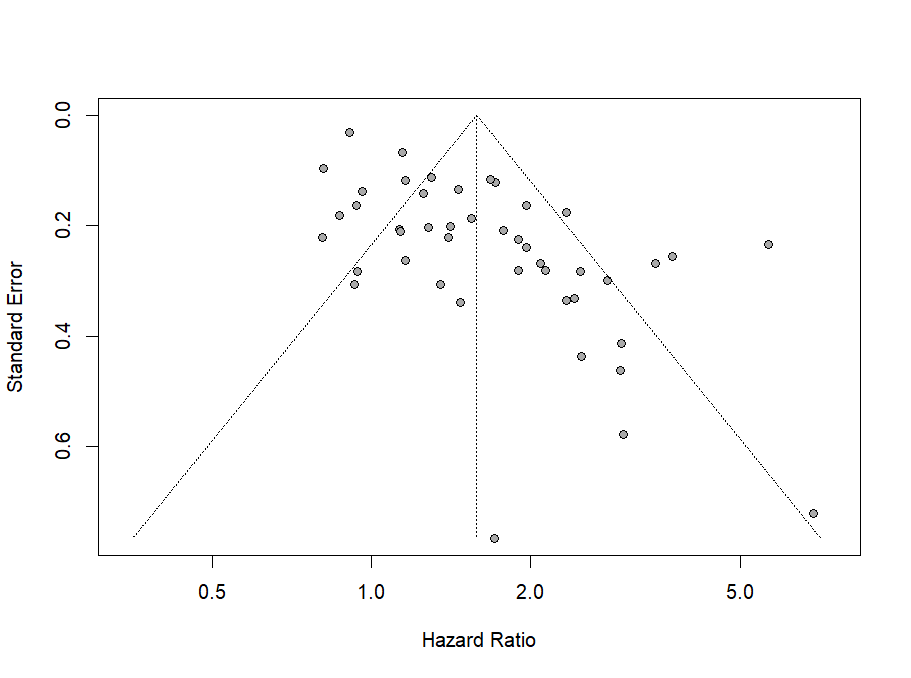

Supplement: Supplementary file 1 [file cancers-17-00607-s001.zip › Supplementary File S26. Funnel plot OS_UNI.png]

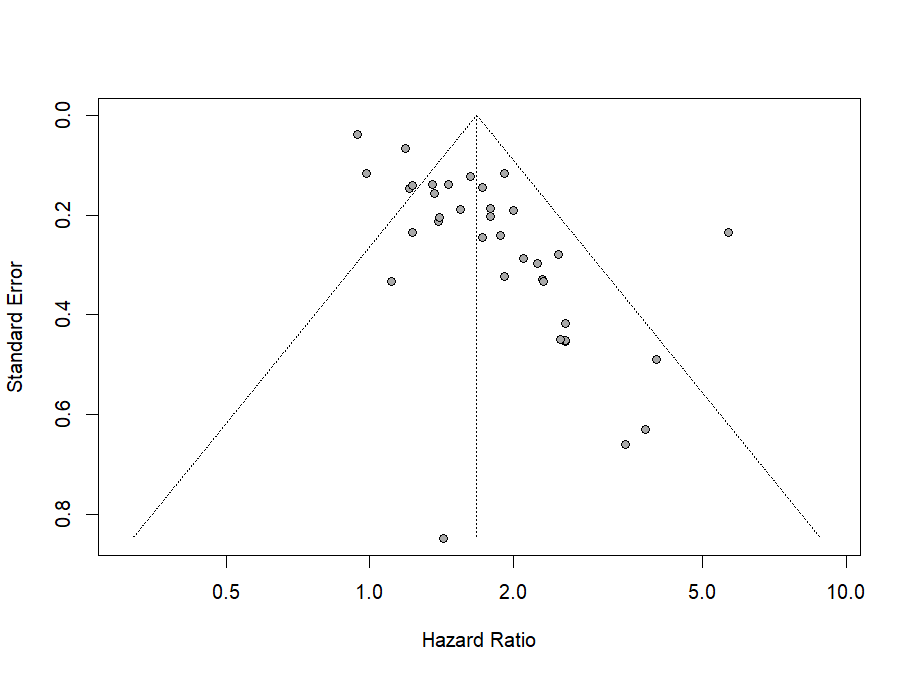

Supplement: Supplementary file 1 [file cancers-17-00607-s001.zip › Supplementary File S27. Funnel plot OS_MULTI.png]

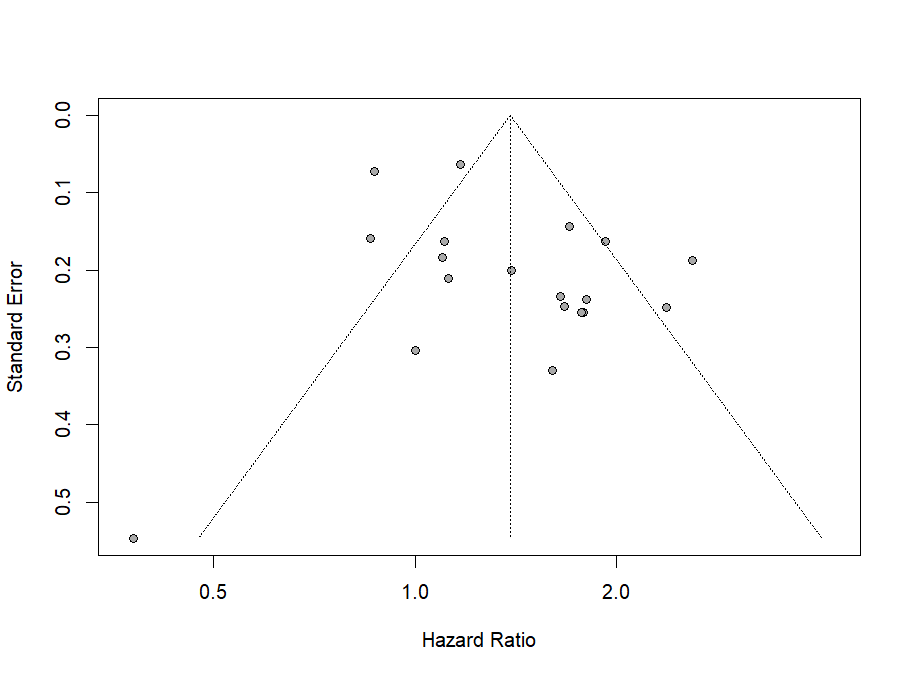

Supplement: Supplementary file 1 [file cancers-17-00607-s001.zip › Supplementary File S28. Funnel plot PFS_UNI.png]

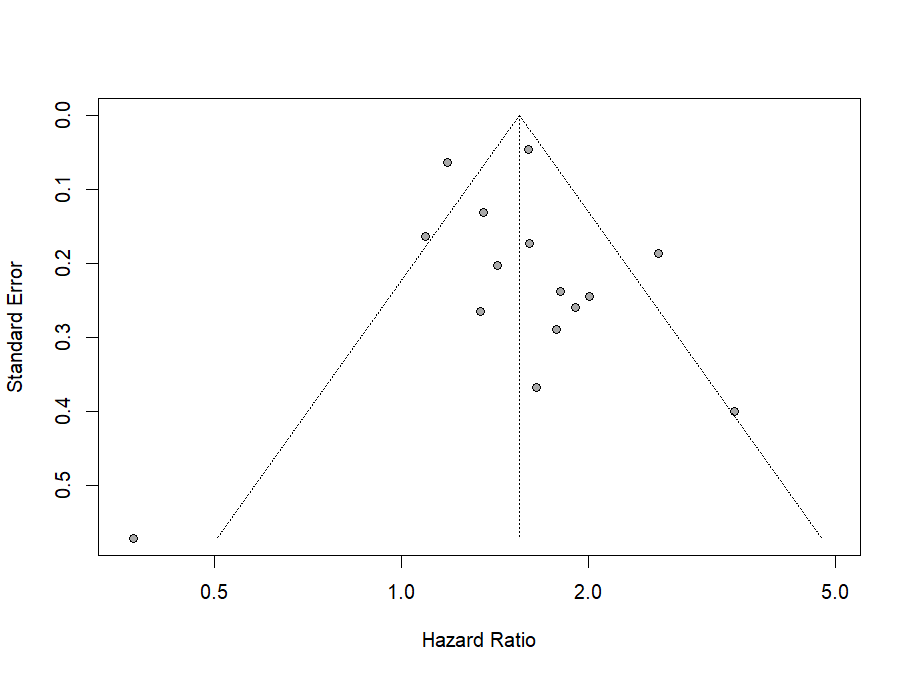

Supplement: Supplementary file 1 [file cancers-17-00607-s001.zip › Supplementary File S29. Funnel plot PFS_MULTI.png]

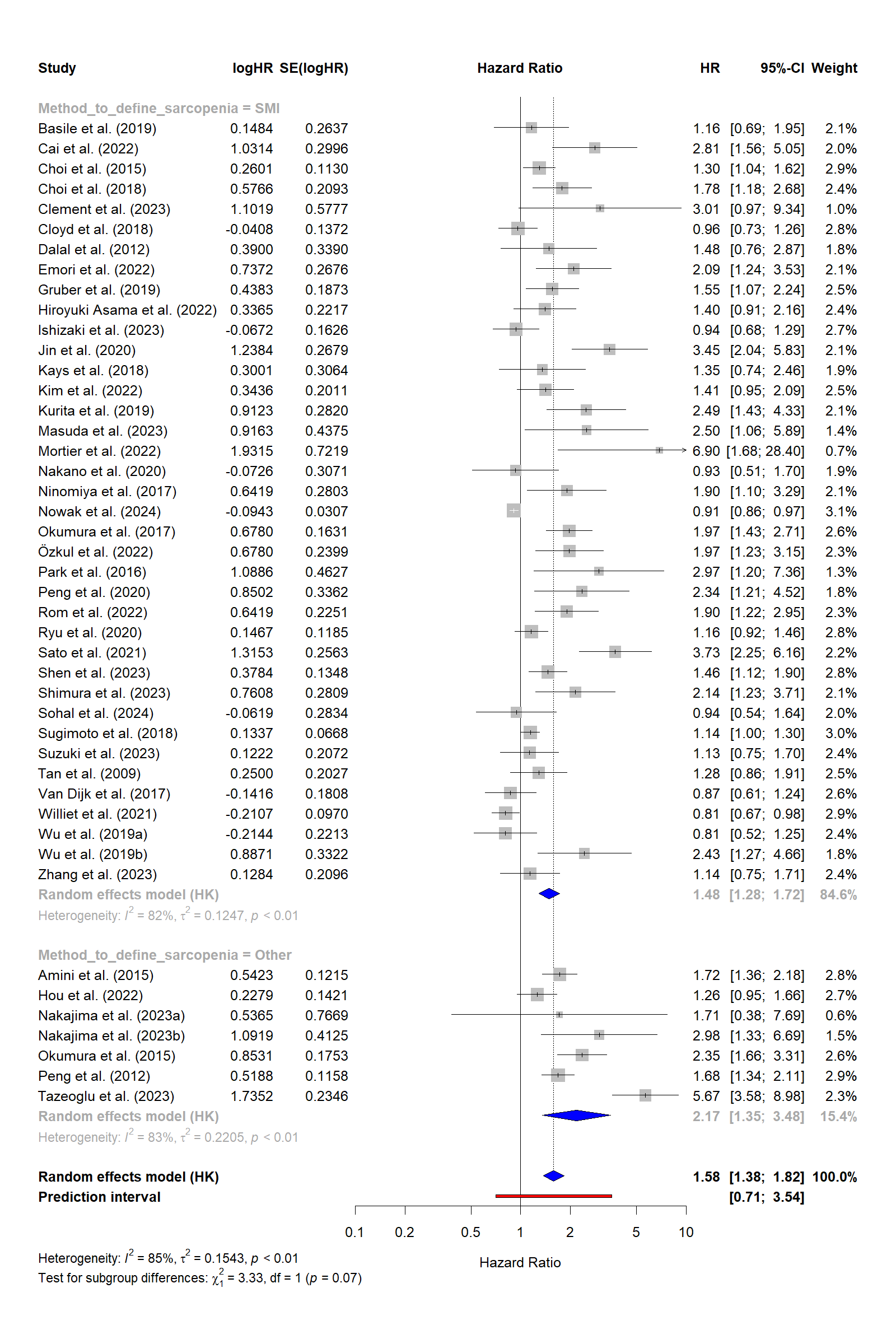

Supplement: Supplementary file 1 [file cancers-17-00607-s001.zip › Supplementary File S6. Forest plot_OS_UNI_subgroup_method.png]

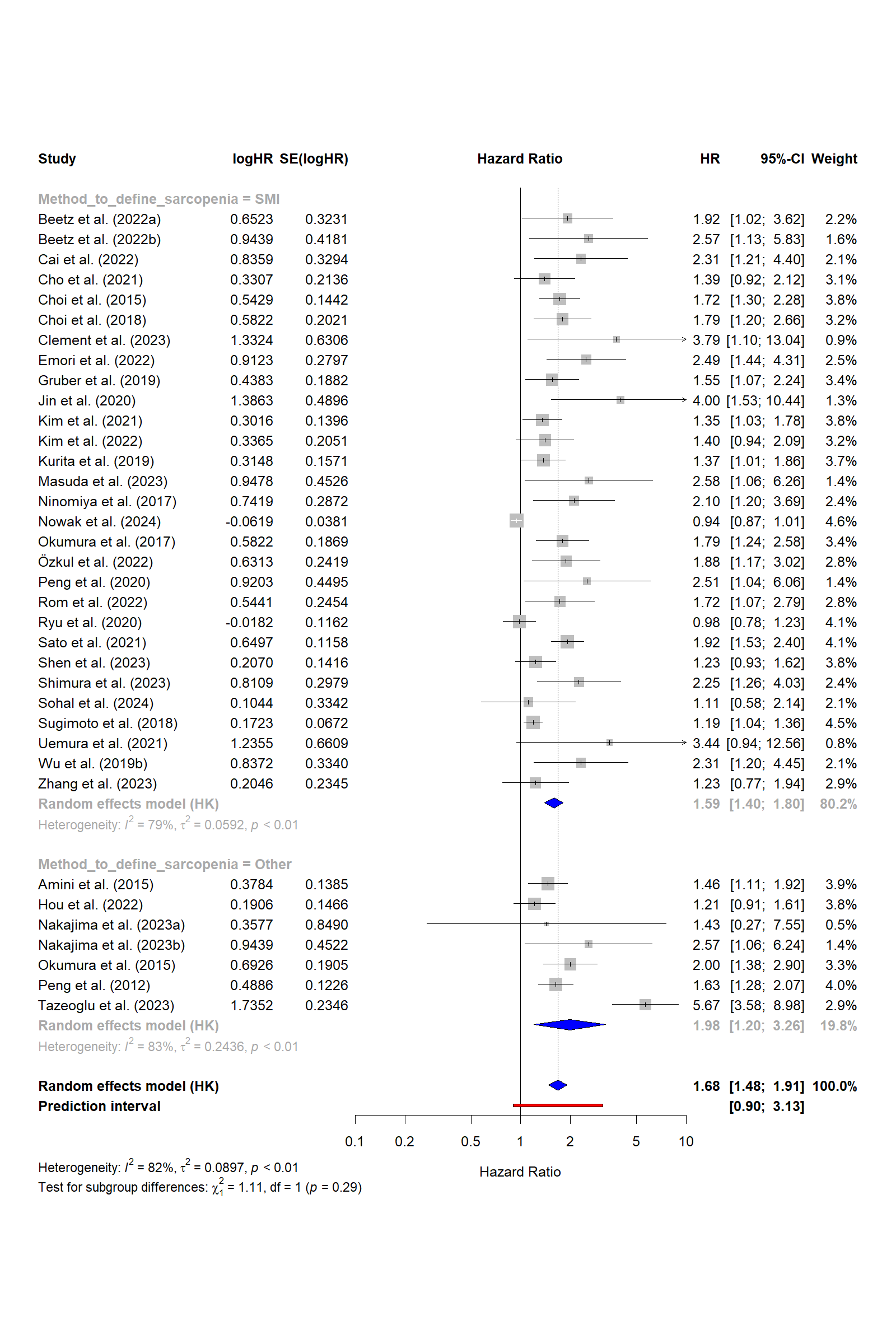

Supplement: Supplementary file 1 [file cancers-17-00607-s001.zip › Supplementary File S7. Forest_plot_OS_MULTI_subgroup_method.png]

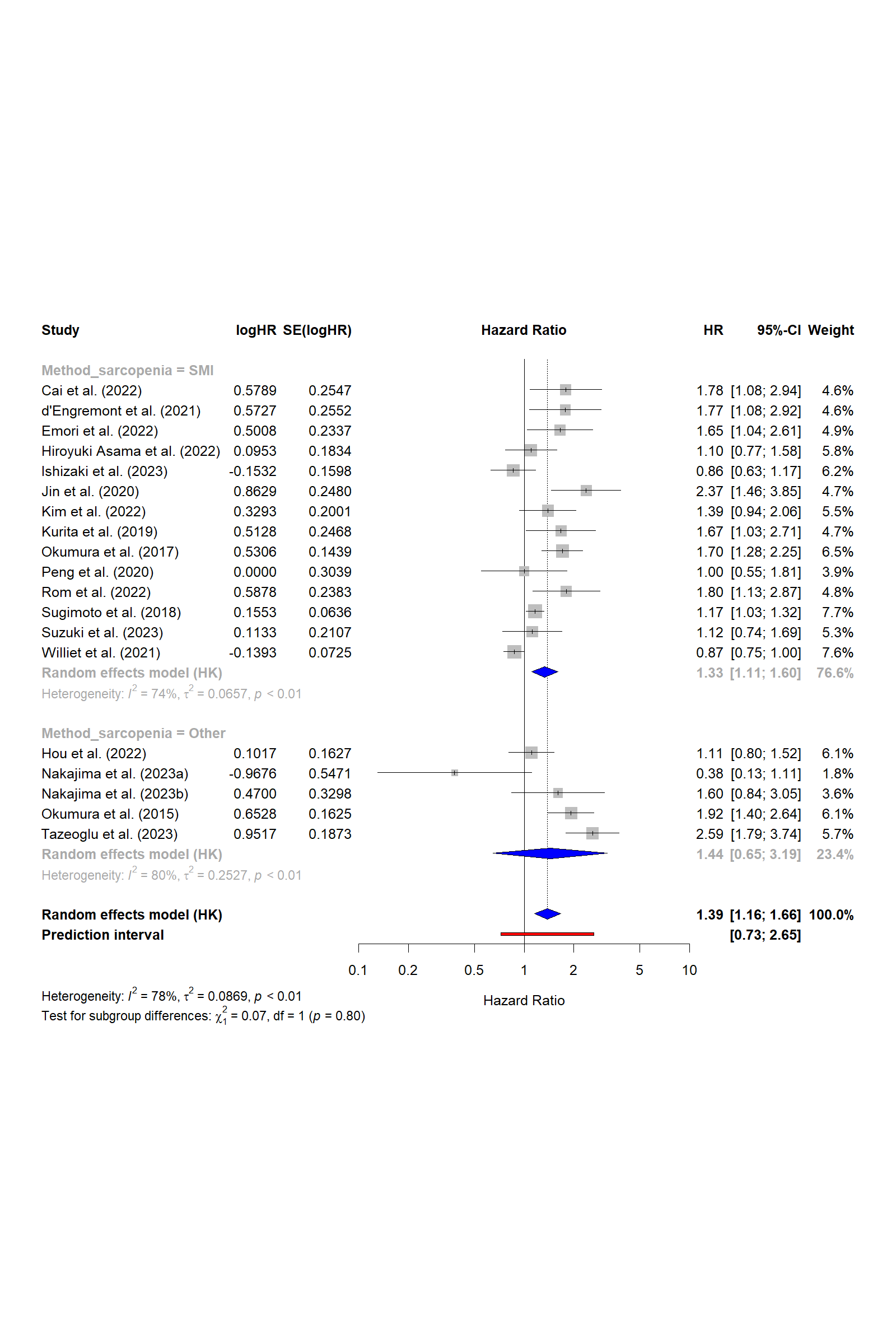

Supplement: Supplementary file 1 [file cancers-17-00607-s001.zip › Supplementary File S8. Forest_plot_PFS_UNI_subgroup_method.png]

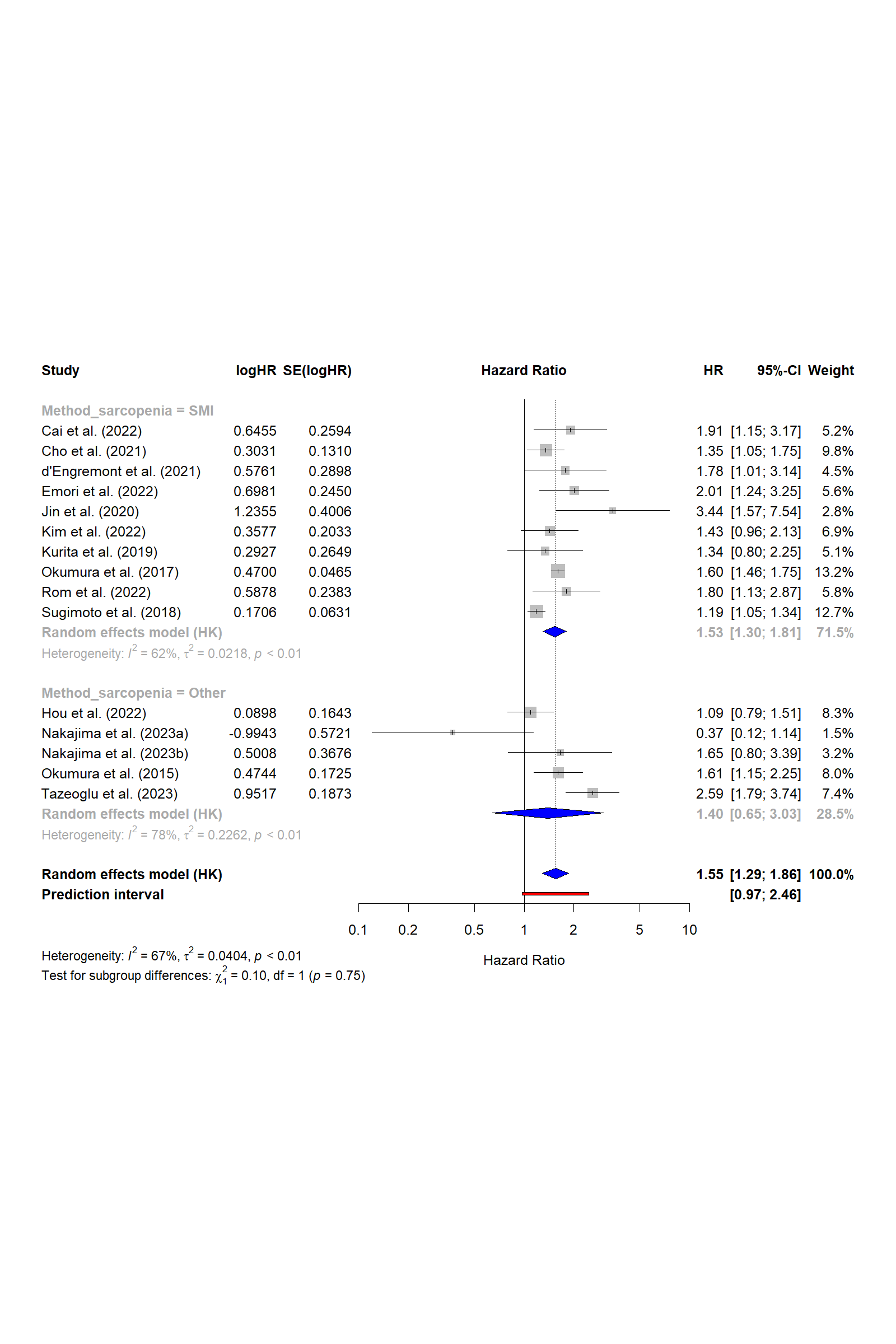

Supplement: Supplementary file 1 [file cancers-17-00607-s001.zip › Supplementary File S9. Forest_plot_PFS_MULTI_subgroup_method.png]
